# Palladium nanoparticles supported on chitin-based nanomaterials as heterogeneous catalysts for the Heck coupling reaction

**DOI:** 10.3762/bjoc.16.201

**Published:** 2020-10-07

**Authors:** Tony Jin, Malickah Hicks, Davis Kurdyla, Sabahudin Hrapovic, Edmond Lam, Audrey Moores

**Affiliations:** 1 Department of Chemistry, McGill University, Montreal, Quebec H3A 0B8, Canada; 2 Aquatic and Crop Resource Development Research Centre, National Research Council of Canada, Montreal, Quebec H4P 2R2, Canada; 3 Department of Mining and Materials Engineering, McGill University, Montreal, Quebec H3A 0E9, Canada

**Keywords:** chitin, chitosan, Heck coupling, heterogeneous catalysis, nanomaterial

## Abstract

In this report, chitin and chitosan nanocrystals were used as biomass-based supports for Pd nanoparticles (NPs) used as a heterogeneous catalyst for the Heck coupling reaction. By using a one-pot fabrication method, a Pd salt precursor was directly reduced and deposited onto these nanocrystal catalysts. Characterization of these nanocomposites showed disperse Pd NPs on the surfaces of the chitinous nanocrystals. Heck coupling model reactions revealed full product yield in relatively benign conditions, outcompeting the use of other catalysts supported on biomass-based nanomaterials, including cellulose nanocrystals. These initial results show the potential for using chitinous nanomaterials as effective catalyst supports in cross-coupling reactions.

## Introduction

Over the past decades, biomass-based nanomaterials have become a highly prevalent topic of research owing to their sustainability, bioavailability, unique structural and morphological characteristics [1]. Particularly dominant in this field are cellulose nanocrystals (CNCs), which are rod-like nanocrystallites liberated from lignocellulosic biomass under acid hydrolysis conditions [2]. A spectrum of applications have been investigated over the years for this sustainable bio-nanomaterial including drug delivery [3], food packaging [4], environmental remediation [5], and catalysis [6]. With their high solubility and presence of functionalities such as hydroxy groups, sulfate half-esters, and carboxylates, CNCs are able to stabilize highly disperse metal nanoparticles (NPs), which can act as heterogeneous catalysts for a wide array of organic transformations [7–9]. Furthermore, the chiral nature of polysaccharides has also been used as a tool for enantioselective catalysis such as carbonyl hydrogenations and amino acid hydrolysis, proving the unique ability of these biomass-based supports [10–11].

Chitin is another type of biomass feedstock that has attracted similar attention to cellulose. Found primarily in squid, insects, fungi, and the shells of crustaceans (shrimp, crab, and lobster), chitin is the second-most abundant biopolymer after cellulose, with an annual availability of over 6 million tons from crustacean shell waste alone [12]. With shell waste being often discarded back into the sea or in landfills, it is imperative that downstream applications be developed such that environmental concerns and disposal costs for this neglected resource are reduced through the creation of bio-based sustainable technologies [13]. In this manner, strategies for fabricating CNCs have been adapted for chitin nanocrystals (ChNCs). From the seminal discovery of ChNCs by Marchessault in 1959 [14], much work has been done to improve the monodispersity, morphology, and structure of this unique nanomaterial [15]. Very recently, we have reported the use of ammonium persulfate as a mild oxidizing agent to liberate the nanocrystallites existing within bulk chitin to yield ChNC with carboxylate functionalities [16]. Moreover, deacetylation of ChNCs in alkaline conditions, in the presence of NaBH_4_, led to chitosan nanocrystals (ChsNCs) with abundant amine groups.

While groups are starting to investigate the usage of chitin and chitosan-based supports for heterogeneous catalysis, there are still scarce investigations on using these biomaterials on the nanoscale, which can allow for higher accessibility of their functionalities towards better stabilizing dispersed metal nanoparticle catalysts, along with increased solubility in aqueous media. Very recently, we have shown that these bio-based nanomaterials could stabilize highly disperse Au species on the surface of these nanocrystals to create a highly active catalyst for aromatic nitro reduction and aldehyde–amine–alkyne (A^3^) coupling reactions [16]. Off this discovery, in this letter, we further expand the scope of using both ChNCs and ChsNCs as a catalyst support for Pd NPs to allow access towards other highly relevant C–C bond-forming reactions. A one-pot fabrication method is used to deposit Pd NPs directly onto both ChNCs and ChsNCs, and the as-made heterogeneous catalysts were tested with the Heck coupling reaction as a model for catalytic activity.

## Findings

**Scheme 1:**
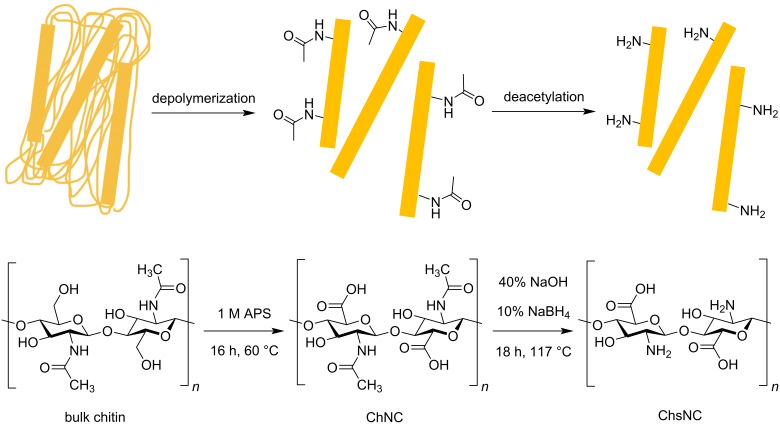
Pathway for the formation of ChNC and subsequently ChsNCs from bulk chitin.

The fabrication of ChNC and ChsNCs was conducted using a procedure previously reported by our group (Lam) (see Supporting Information File 1) [16]. ChNCs were treated with ammonium persulfate (APS) for 16 h to form disperse ChNCs after washing. ChsNCs were made by deacetylating ChNCs in the presence of concentrated NaOH as well as a small amount of NaBH_4_ (Scheme 1).

**Figure 1:**
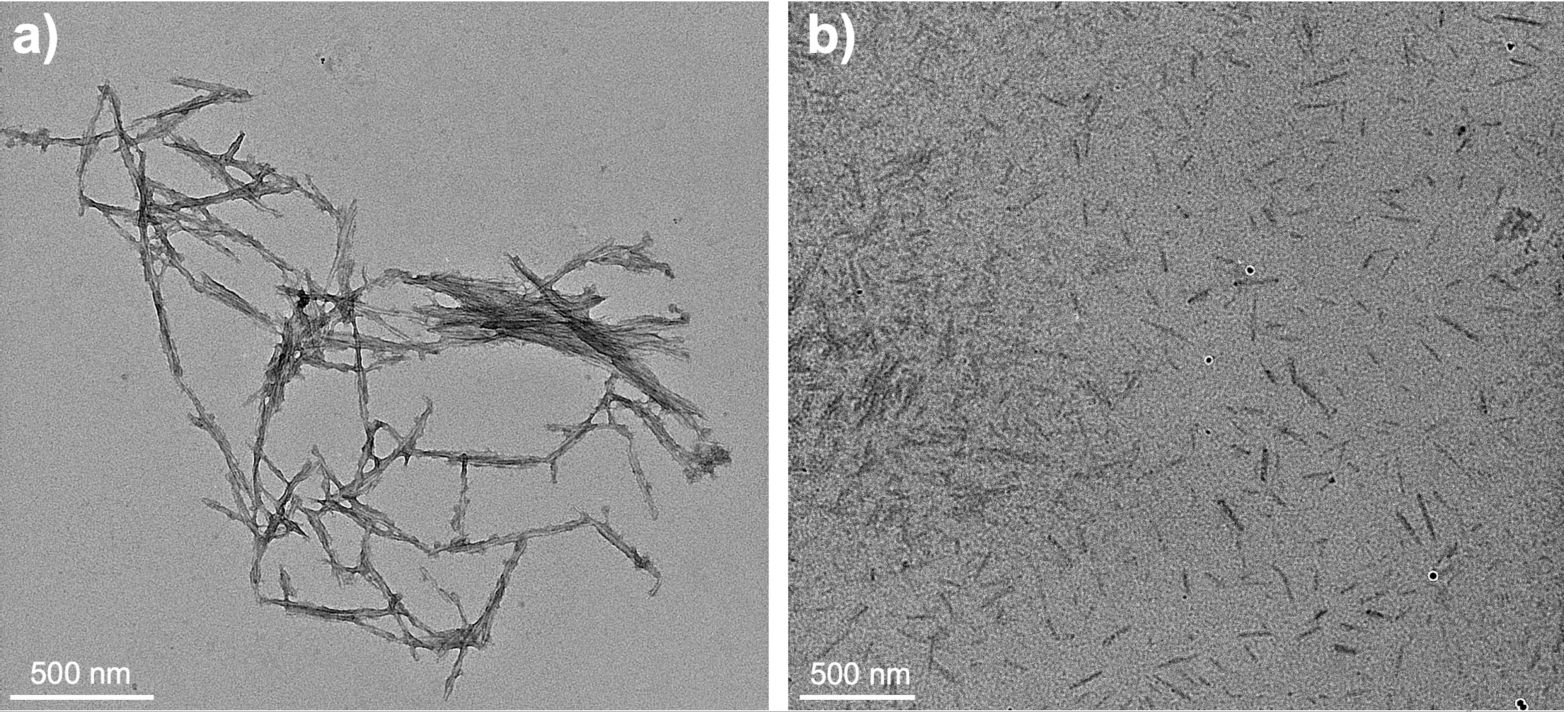
TEM micrographs of (a) ChNCs and (b) ChsNCs. Both samples were stained and prepared on glow-discharged C-coated Cu TEM grids.

As seen through transmission electron microscopy (TEM) in Figure 1, a relatively uniform distribution for both ChNCs and ChsNCs with individual rod-like nanocrystals was observed, with an average length of 231 ± 38 nm for the ChNC and 159 ± 34 nm for the ChsNC (Supporting Information File 1, Figure S1). These measures were made from parts in the grid where the nanocrystals were well separated. Larger aggregates of the individual nanocrystals were also observed in all samples. Glow-discharged carbon-coated TEM grids were used, along with uranyl acetate as a negative stain in order to provide higher contrast to the individual rods.

Further characterization of the ChNCs and ChsNCs confirmed the structural and chemical functional properties of these nanomaterials. We turned to Fourier transform infrared (FTIR) spectroscopy to access the degree of deacetylation (DDA) of the prepared materials (Supporting Information File 1, Figure S2). The ratio of primary amine over the sum of nitrogen-containing functionalities can be derived through the measurement of the N–H bend and C–O stretch peaks absorbance, found at 1560 cm^−1^ and 1030 cm^−1^, respectively [17]. In general, bulk chitin has a DDA of 0–20%, while chitosan has a DDA of >80% [18]. The fabricated ChNCs had DDA values of 5–10%, while the ChsNCs had DDA values between 80–95%. A full spectral assignment for all of the FTIR peaks can be found in our recent report [16].

This transformation of the acetamide functionality into an amine one had drastic effects on the physicochemical properties of the nanomaterials. Specifically, the deacetylation of ChNC into ChsNC led to a decrease in crystallinity in the nanomaterial. Indeed, this can be seen in the FTIR with the broadening of the N–H and O–H stretches from 3000–3500 cm^−1^. This was more notable in the powder X-ray diffraction (PXRD) spectra of ChNCs and ChsNCs (Supporting Information File 1, Figure S3) where broadening of the peaks was observed for ChsNC as amorphization of the internal ChNC structure occurred during the deacetylation process. ChsNCs were readily suspendable in aqueous media and formed a transparent solution, owing to their positively charged amino functionality, while ChNCs were less easily suspended. Zeta potential measurements of −24.6 mV for ChNCs and +36.8 mV for ChsNCs provided a rationale for these observations. With these differences between the two nanomaterials, we then explored how they behaved as catalyst supports for Pd NPs.

**Scheme 2:**
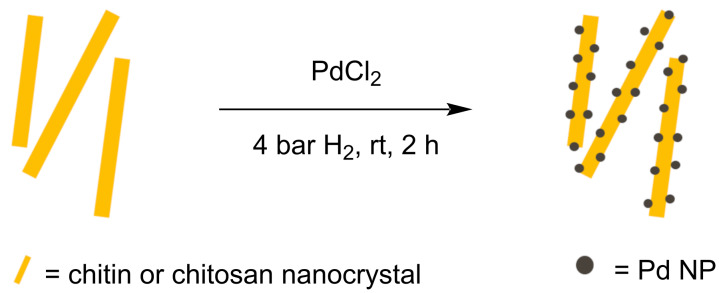
Catalyst fabrication method for the deposition of Pd NPs onto chitin (PdNP@ChNC) and chitosan (PdNP@ChsNC).

A one-pot synthesis method was used to both deposit Pd salts and reduce them into NPs onto the support material. First, PdCl_2_ was mixed for 15 min with either ChNC or ChsNC in an acidic aqueous medium to form a dark yellow mixture. This step facilitated coordination of Pd salts onto the support, as evidenced when using CNC as support [7]. From their synthesis involving oxidative conditions, both ChNC and ChsNC featured carboxylate functionalities on their surface which we expected to be good chelating functionalities for Pd(II) (Scheme 1) [16]. For ChsNC, amines were unlikely to play any coordinating role, since they should be fully protonated under acidic conditions. Then, the mixtures were subjugated to 4 bar H_2_ for 2 h at room temperature to reduce Pd(II) into metallic Pd NPs (Scheme 2), and the reaction mixture turned black. We also conducted a control study in the exact experimental parameters were performed on PdCl_2_ and either ChNC or ChsNC, but with no H_2_ reductant. In this case, the solution color remained yellow, indicating that using either ChNC or ChsNC alone cannot fully reduce PdCl_2_. The resulting hybrid materials are noted PdNP@ChNC and PdNP@ChsNC, respectively.

**Figure 2:**
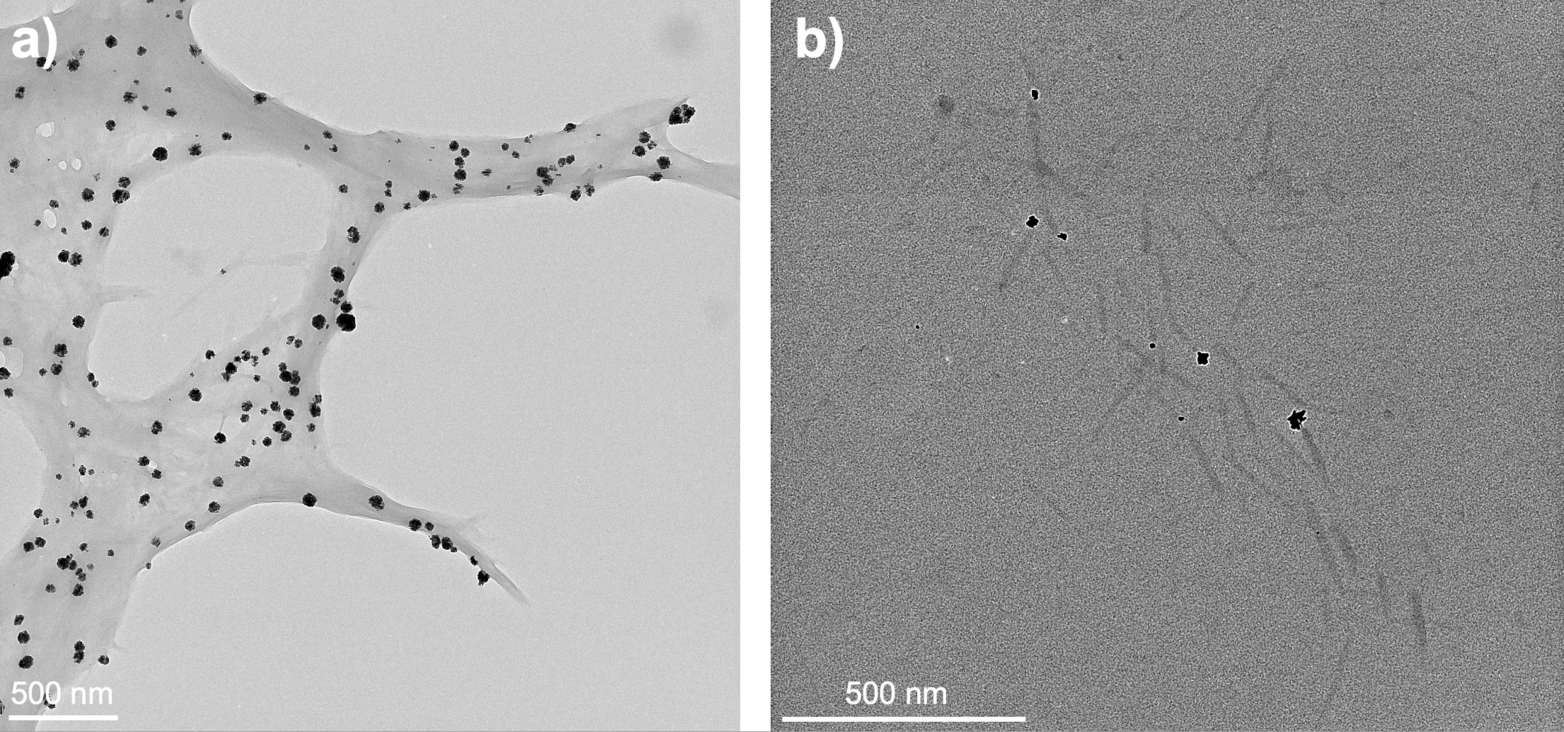
TEM micrographs of (a) PdNP@ChNCs and (b) PdNP@ChsNCs. The samples were placed on glow discharged TEM grids, but unstained. The images were taken purposefully with high contrast and large objective aperture to capture the nanocrystals.

Dihydrogen was selected here because it is one of cleanest reductants in this context as it will limit the production of byproducts to chloride salts, by opposition to more classic reducing agents such as NaBH_4_. Prior to characterization, the non-dried samples were purified by dialysis. The zeta potential measurements for PdNP@ChNC and PdNP@ChsNCs were −13.9 and +57.9 mV, respectively. PdNP@ChsNCs were, again, far more suspendable in aqueous solution as compared to PdNP@ChNCs. TEM micrographs of PdNP@ChNC (Figure 2a) and PdNP@ChsNC (Figure 2b) confirmed complete immobilization of Pd NPs onto both the ChNC and ChsNC, with energy dispersive X-ray (EDX) spectroscopy confirming the presence of Pd (Supporting Information File 1, Figure S4). PdNP@ChNCs self-aggregated while drying during the TEM sample preparation procedure, despite the use of glow discharged TEM grids. Conversely, the PdNP@ChsNCs were dispersed owing to the higher solubility of ChsNC. Both PdNP@ChNCs and PdNP@ChsNCs were imaged unstained to avoid any artefact in Pd imaging [19]. Dispersed “packets” of Pd NPs were observed for both samples, with far more packets observed for PdNP@ChNC (packet diameter of 42 ± 10 nm) samples compared to PdNP@ChsNC (packet diameter of 24 ± 7 nm) samples for the same wt/wt loading of the PdCl_2_ salt to ChNC/ChsNC (initially set to 1.6 wt %). It is also noted that the packets found in PdNP@ChsNC were almost half as small relative to PdNP@ChNC. At higher magnification, these packets are seen to be extremely small Pd NPs agglomerated together (Supporting Information File 1, Figure S5). A similar packet formation was observed when the wt/wt loading of PdCl_2_ was reduced by to 0.8 wt % to fabricate PdNP@ChNC (Supporting Information File 1, Figure S6).

**Figure 3:**
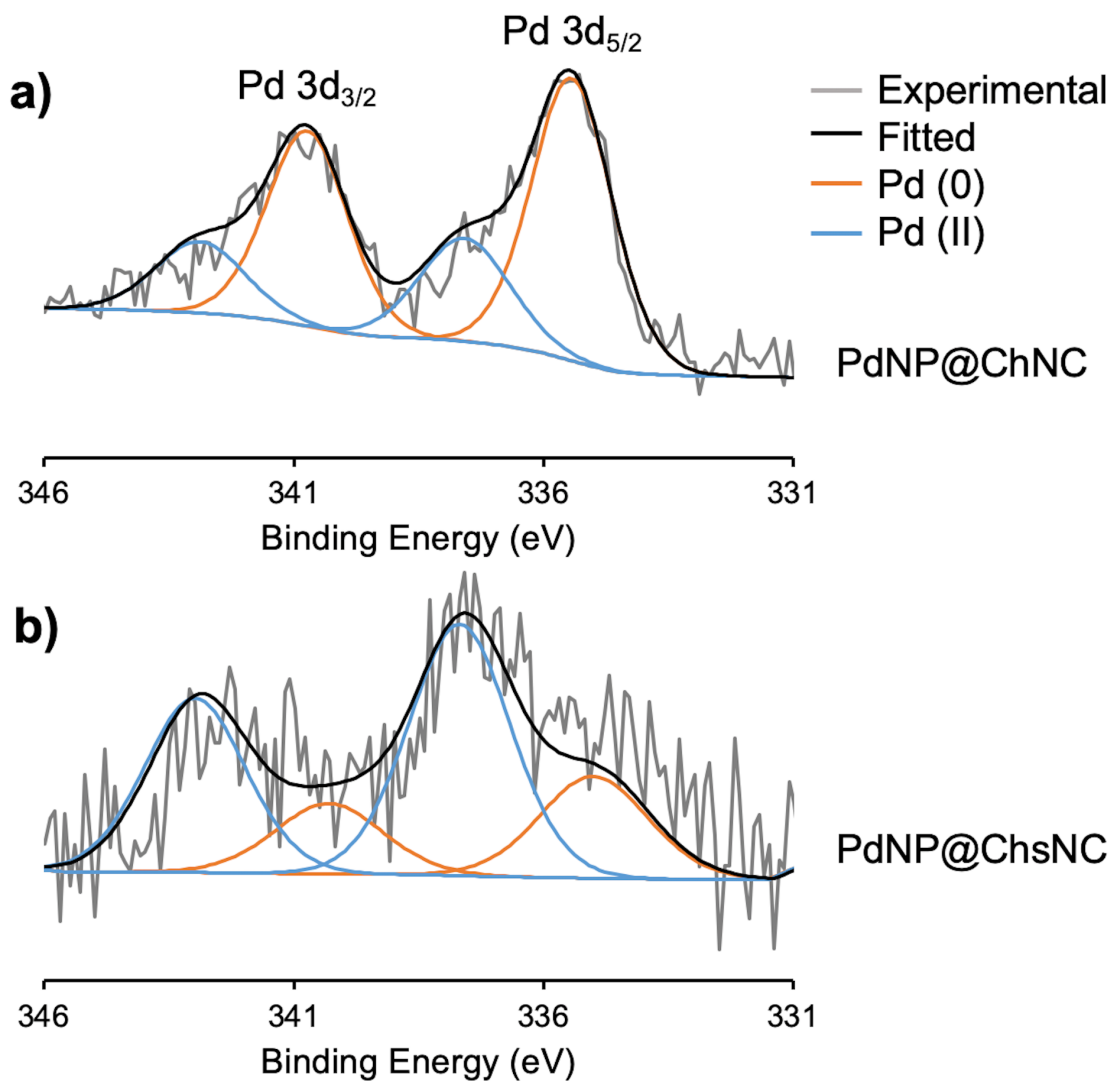
High-resolution X-ray photoelectron spectroscopy of the Pd 3d region of (a) PdNP@ChNC and (b) PdNP@ChsNC. Deconvolution of the experimental peaks (grey line) of each spectrum with a fitted (black line) leads to the Pd(0) (orange line) and Pd(II) (blue line) doublets.

X-ray photoelectron spectroscopy (XPS) was used to confirm the oxidation state of Pd on both the support materials (Figure 3). The experimental XPS spectra were deconvoluted and their match with thus obtained fitted data confirmed. Firstly, Pd on PdNP@ChNC was mainly Pd(0), with the Pd 3d_5/2_ peak residing at 335.1 eV, along with a small shoulder at higher binding energy indicating the presence of Pd(II) (Figure 3a). In contrast, Pd primarily exists as Pd(II) on PdNP@ChsNC (Figure 3b), which could be attributed to three possible reasons: 1) the presence of PdCl_2_, 2) the oxidation of Pd NPs into PdO, and 3) the complexation of Pd(II) by ChsNCs. To address the first point, a survey XPS scan showed no Cl contribution to the overall atom distribution suggesting that no PdCl_2_ species were present in the nanocomposites. For the second point, high-resolution XPS spectra on the O 1s scan of both the PdNP@ChNC and PdNP@ChsNC samples show the exact same symmetric peak, similar to that of bare ChNC and ChsNC, indicating that no formation of a Pd–O bond is present (Supporting Information File 1, Figure S7). A more accurate explanation is through the third point where Pd(II) is present on ChsNC over ChNC, an observation further validated by TEM micrographs that showed consistently fewer metallic Pd NPs on ChsNC than ChNCs. This point is corroborated by the very significant increase in zeta potential from +36.8 mV to +57.9 mV from ChsNCs to PdNPs@ChsNCs, which can only be explained by the integration at the nanocrystals surface of positively charged species, namely Pd(II). This resistance to reduction was surprising and in contradiction to what we observed with deposition onto CNC of Pd in the presence of H_2_ [7], or Ag alone [20]. The striking difference between CNC on one hand and ChNC/ChsNC on the other is the presence of carboxylates on the latter. Carboxylates are expected to afford much stronger coordination to Pd(II) than OH typically present in CNC, and potentially prevent its full reduction.

Through FTIR (Supporting Information File 1, Figure S2) and XRD (Supporting Information File 1, Figure S3) analysis, there is little to no structural changes occurring in either the ChNC or the ChsNC during catalyst fabrication. The lack of metallic Pd peaks present in XRD is indicative of extreme broadening of the reflections of very small Pd NPs within the packets found.

Heck coupling is a prominent reaction for arene alkenylation, as the production of stilbene derivatives is highly relevant in areas of research such as pharmaceuticals and materials technology [21]. Furthermore, works in heterogeneous catalysis have shown that the catalyst support plays a major role in the activity of transition-metal NPs such as Pd [7]. A model reaction was performed under the conditions listed in Table 1, at 90 °C for 24 h using only PdNP@ChNC at 1 mol % Pd relative to iodobenzene. Full product yield was achieved, and another replicate was done for accuracy (Table 1, entry 1). However, a decrease in the reaction temperature to 70 °C yielded virtually no product (Table 1, entry 2). By keeping the system at 90 °C and shortening the time to 6 h, 35% product yield was already obtained, indicating a promising reaction rate (Table 1, entry 3). If the Pd loading was lowered to 0.5 mol %, a stark drop in the yield was observed (Table 1, entry 4). In entry 5 (Table 1), PdNP@ChNC with a lower Pd wt % relative to ChNC was used which yielded a lower product yield of 52% despite retention of 1 mol % loading relative to iodobenzene. The ChNC is potentially hindering the ability for substrates to interact with the Pd sites at such low wt % of Pd on the surface of the ChNC support.

**Table 1:** Heck coupling reaction optimization.^a^

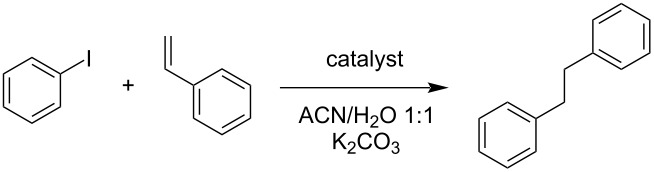				

entry	catalyst	time (h)	temperature (°C)	yield^b^

1	PdNP@ChNC	24	90	100
2	PdNP@ChNC	24	70	<1
3	PdNP@ChNC	6	90	35
4	PdNP@ChNC^c^	24	90	38
5	PdNP@ChNC (0.8 wt % Pd)^d^	24	90	52
6	PdNP@ChsNC	24	90	3
7	ChNC^e^	24	90	0
8	PdCl_2_ and ChNC	24	90	43

^a^All reactions listed used 0.2 mmol of iodobenzene and 0.24 mmol of styrene, and a Pd loading relative to iodobenzene of 1 mol %, unless otherwise specified. The solvent was 1:1 acetonitrile/water. ^b^Yield was determined through GC–MS with hexamethylbenzene as an internal standard (Supporting Information File 1, Figure S8). ^c^Reaction done with 0.5 mol % Pd loading relative to iodobenzene. ^d^Reaction done with PdNP@ChNC using 0.8 wt % Pd relative to ChNC, as opposed to 1.6 wt % like the standard PdNP@ChNC. ^e^Pd loading is 0 mol %.

In contrast to PdNP@ChNC, PdNP@ChsNC showed little product yield in the model reaction (Table 1, entry 6), which was surprising as our previous works showed that ChsNC was the superior catalyst support for Au-catalyzed A^3^ coupling reactions [16]. The XPS analysis of PdNP@ChsNC suggests that Pd is complexed to ChsNC in the +2 oxidation state (Figure 3). These results align with our previous work where ChsNCs tend to stabilize Au in the +1 oxidation state as opposed to metallic Au. Since the Heck coupling primarily follows a classic oxidative addition/reductive elimination pathway with Pd(0) being the active catalytic site in most cases [22], Pd(II) would be inactive towards oxidative addition of the electrophilic iodobenzene, leading to no product formation. Importantly, even if mild reducers were present to initiate the cycle and afford Pd(0), as is often the case in Pd cross coupling chemistry, the fact that 4 bar H_2_ pressure was not able to reduce these species is a strong indication of their stability against reduction. We also tested the controls to show that the ChNC support alone was catalytically inactive (Table 1, entry 7). We also demonstrated that direct mixing of PdCl_2_ with ChNC had minor catalytic effect (Table 1, entry 8), likely because the partial reduction taking place under these conditions was ineffective in affording the well-defined nanoparticles we synthesized as PdNPs@ChNC.

Comparisons within the literature were made with similar Pd NP-based systems (Table S1 in Supporting Information File 1). Firstly, it can be seen that the PdNP@ChNC system outcompetes a similar system with Pd NP on CNCs, which led to 75% yield in 24 h and 100 °C, albeit with lower Pd loading [7]. Further comparisons with Pd NPs on other supports such as SiO_2_ as reported by Jadhav et al. also suggest our system has higher catalytic activity in more benign conditions, with the Pd NP on SiO_2_ system yielding 92% stilbene product at 110 °C and using dimethylformamide as the solvent [23]. Other examples using carbon-based supports such as carbon spheres [24] and graphene oxide [25] also have formidable yields, yet with either very high temperatures greater than 100 °C or using organic solvents such as toluene. The comparison with recent work in using chitin microspheres shows that chitin-based supports are potentially valuable support materials, with full product conversion in only 10 h, yet with mostly organic solvents (4:1 DMF/H_2_O) [26].

## Conclusion

ChNCs and ChsNCs were explored as sustainable supports for immobilizing Pd NPs to fabricate heterogeneous catalysts for the Heck coupling reaction. Through TEM and XPS analysis, metallic Pd NPs were formed and dispersed on the surface of the supports, while FTIR and PXRD showed little to no structural change to the biomaterials after metal deposition. Heck coupling results demonstrate the importance of using ChNCs as opposed to ChsNCs in order to control the redox chemistry of Pd, with full product yield in relatively mild conditions using PdNP@ChNC.

## Supporting Information

Supporting Information features experimental procedures depicting the materials used, the syntheses of ChNC and ChsNC, fabrication methods for PdNP@ChNC and PdNP@ChsNC, the standard reaction protocol for Heck coupling, characterization information as well as additional characterization such as FTIR, PXRD, and supplemental TEM images.

File 1 Experimental part.
