# Supplementary material for: Palladium nanoparticles supported on chitin-based nanomaterials as heterogeneous catalysts for the Heck coupling reaction
Source: Beilstein J Org Chem. 2020 Oct 7;16:2477–83. doi: 10.3762/bjoc.16.201 (PMC7554660; doi:10.3762/bjoc.16.201)
Supplement: File 1 — Experimental part. [file Beilstein_J_Org_Chem-16-2477-s001.pdf]

BEILSTEIN JOURNAL OF ORGANIC CHEMISTRY

Supporting Information

for

Palladium nanoparticles supported on chitin-based nanomaterials as heterogeneous catalysts for the Heck coupling reaction

Tony Jin, Malickah Hicks, Davis Kurdyla, Sabahudin Hrapovic, Edmond Lam and Audrey Moores

Beilstein J. Org. Chem. **2020,** *16,* 2477–2483. doi:10.3762/bjoc.16.201

Experimental part

License and Terms: This is a supporting information file under the terms of the Creative Commons Attribution License (https://creativecommons.org/licenses/by/4.0). Please note that the reuse, redistribution and reproduction in particular requires that the authors and source are credited.
The license is subject to the *Beilstein Journal of Organic Chemistry* terms and conditions: (https://www.beilstein-journals.org/bjoc)

Materials
All reagents and solvents were of reagent grade and used as received. Water used was Milli-Q 18 MΩ. Chitin (from shrimp shells), iodobenzene, and styrene were obtained from Sigma Aldrich. Ammonium persulfate was obtained from FMC Corp. (Philadelphia, PA). Palladium chloride was obtained from Pressure Chemicals (Pittsburgh, PA).

ChNC/ChsNC Synthesis
Both ChNCs and ChsNCs were prepared using a procedure adapted previously reported by our group [1]. Shrimp chitin (27.2 g) was added to ammonium persulfate (APS, 1 M, 540 mL) and stirred at 60 °C for 16 h. The suspension was then centrifuged at 10,000 rpm (RCF = 15,300*g*) for 10 min, and the pellet was washed six times with water. ChNCs (14 g) were isolated via lyophilization as a white solid. ChsNCs were prepared by mixing ChNCs (50 g) and $NaBH_4$ (2.5 g) in NaOH (2.5 L, 40 wt % solution in water) and heated to reflux at 117 °C for 18 h. The suspension was centrifuged at 10,000 rpm for 10 min, the supernatant was decanted, the pellet was resuspended in NaOH (2.5 L, 40 wt % solution in water) and heated under the same conditions. This process was repeated two to three times, centrifuging the suspension followed by resuspending the pellet in fresh NaOH solution. A small sample of solid was taken after each reflux step and the DDA was determined by monitoring the FTIR absorbance peaks at 1030 and 1560 cm^{-1} according to a characterization method outlined by Shigemasa [2]. Once the DDA was above 80%, the ChsNC pellet was suspended in deionized water and centrifuged in the same conditions as above. The centrifugation/washing cycles were repeated eight times until the solution conductivity was below 400 $\mu S \cdot cm^{-1}$. The purified pellet was resuspended in water, and HCl (1 M) was added until the solution became slightly acidic (pH 5). The ChsNC product was lyophilized to yield a white powder (29.0 g, 58% yield).

PdNP@ChNC Synthesis
A ChNC suspension (10 mg mL^{-1}, 25 mL) was mixed with 0.01 M aqueous $PdCl_2$ solution (3.75 mL), followed by addition of HCl until a pH of 2 was achieved for full dissolution of $PdCl_2$. The reaction mixture was stirred for 15 min, before being placed under 4 bar H_2 pressure in a PARR reactor system for 2 h. The solution was subsequently dried under vacuum and lyophilized further to obtain a black powder (91%, 0.226 g) for TEM and XPS, and never dried for use in Heck coupling reactions.

PdNP@ChsNC Synthesis
A ChsNC suspension (10 mg mL^{-1}, 25 mL) was mixed with 0.01 M aqueous $PdCl_2$ solution (3.75 mL) followed by addition of HCl until a pH of 2 was achieved for full dissolution of $PdCl_2$. The reaction mixture was stirred for 10 min, before being placed under 4 bar H_2 pressure in a PARR reactor system for 2 h. The solution was subsequently dried under vacuum and lyophilized further to obtain a black powder (84%, 0.22 g) for TEM and XPS, and never dried for use in Heck coupling reactions.

Characterization
The transmission electron microscopy (TEM) images were taken on a Thermo Scientific Talos F200X G2 S/TEM equipped with a Ceta 16M CMOS camera, with energy dispersive X-ray (EDX) spectroscopy done on a Super-X EDS detector system. Sample preparation for TEM were done on freshly glow-discharged grids using EMS GloQube-D, Dual chamber glow discharge system (Electron Microscopy Sciences, PA) operating in negative mode and applying the plasma current of 25 mA during 45 s. Afterwards, a 10 µL of sample solution was adsorbed to a glow-discharged carbon-coated copper TEM grid (Electron Microscopy Sciences, PA). The partially dried grid was washed three times with distilled water and excess water was removed by carefully blotting the grid with a piece of filter paper. The ChNC and ChsNC samples were subsequently stained twice for 14 s and 45 s with 1% uranyl acetate, then dried at room temperature prior to the TEM observation. The PdNP@ChNC and PdNP@ChsNC samples were not stained.

X-Ray photoelectron spectroscopy (XPS) analyses were conducted on a Fischer Scientific Kα spectrometer using a spot size of 200 µm, running five survey scans at 200 mV for 50 ms residence time, and ten scans for specific elements, also at residence times of 50 ms. Deconvolution and peak position were determined using Avantage processing software.

Fourier transform infrared (FTIR) spectra were collected from 4000 to 400 cm^{-1} for 100 scans at a resolution of 4 cm^{-1} using a Bruker Tensor 27 FTIR spectrometer. Samples were run in the ATR mode over a ZnSe crystal.

X-ray diffraction spectra were acquired using a Bruker D8 Advance X-ray diffractometer equipped with a CuKα filament, scanned with a 2θ range between 5–60° with an increment of 0.02°.

Dynamic light scattering and the zeta potential of the samples (2 mg mL^{-1}) in water were determined using a Zetasizer Nano-ZS (Malvern Instruments, Malvern, UK) in triplicate.

Heck coupling
A 25 mL 14/20 round-bottomed flask was charged with 0.2 mmol of iodobenzene and 0.24 mmol of styrene with 2.5 mL of acetonitrile. Hexamethylbenzene solution in acetonitrile (0.05 M, 0.8 mL) and 0.4 mmol of K_2CO_3 were added to the flask. Lastly, PdNP@ChNC or PdNP@ChsNC solution (1.5 mL) and 1.8 mL of water were added to ensure a solvent ratio 1:1 of acetonitrile/water prior to reaction.

GC–MS analysis
All the GC analyses were performed using an Agilent 7890A series chromatograph equipped with a MS detector (5975C) on an Agilent HP-5 column. After the reaction, the crude mixture was extracted three times with diethyl ether (10 mL) with a separatory funnel and subsequently dried with $MgSO_4$. After vacuum filtration, the filtrate was then diluted with 15 mL of diethyl ether and GC–MS analysis was performed on the sample.

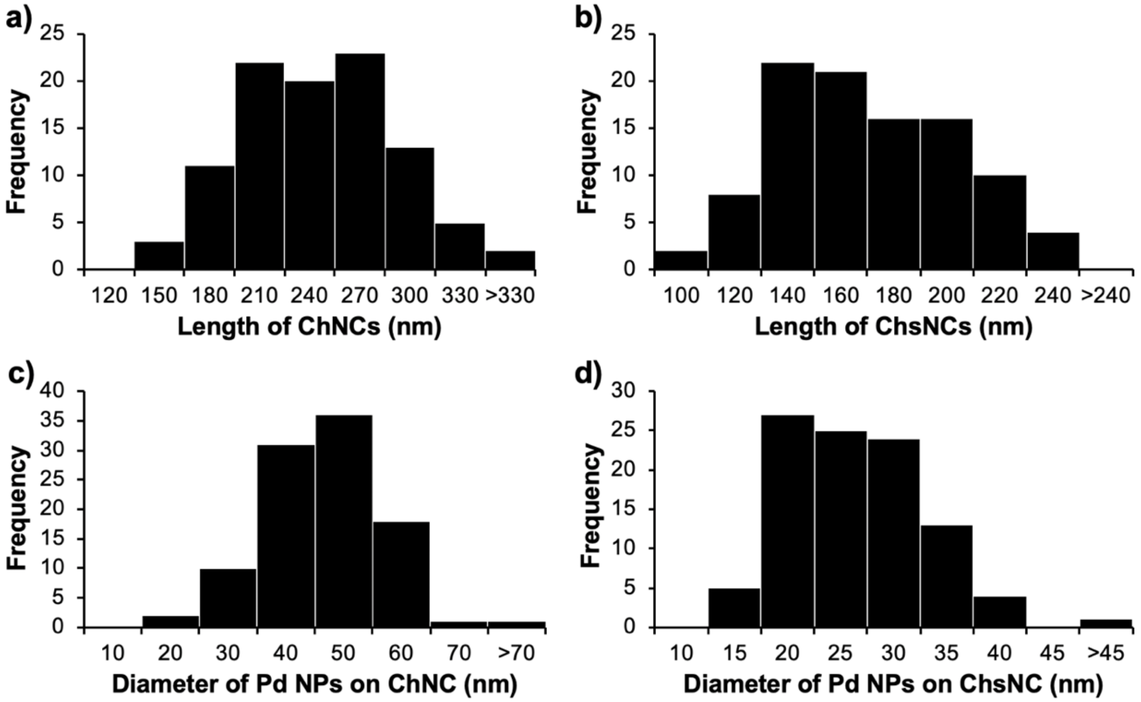

Figure S1: Histograms showing the size distribution for (a) the length of ChNCs, (b) the length of ChsNCs, (c) diameter of Pd NP packets on ChNCs (PdNP@ChNC) and (d) diameter of Pd NP packets on ChsNC.

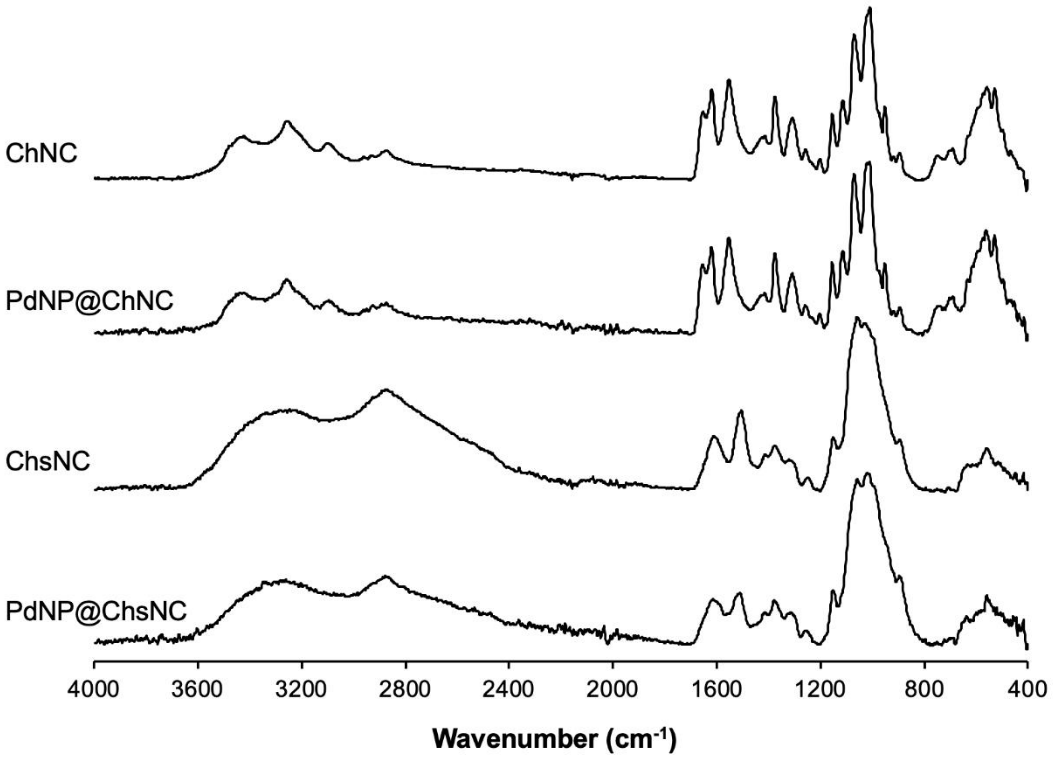

Figure S2: FTIR spectra of ChNC, PdNP@ChNC, ChsNC, and PdNP@ChsNC.

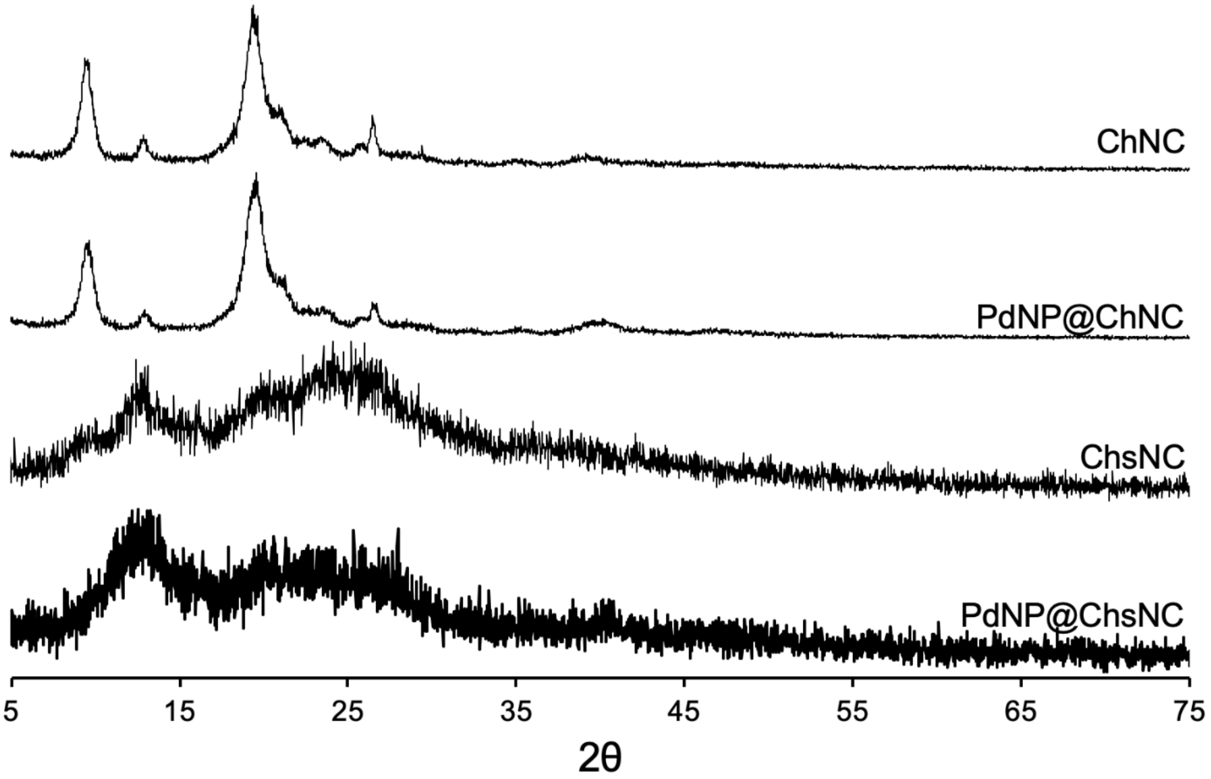

Figure S3: PXRD spectra of ChNC, PdNP@ChNC, ChsNC, and PdNP@ChsNC.

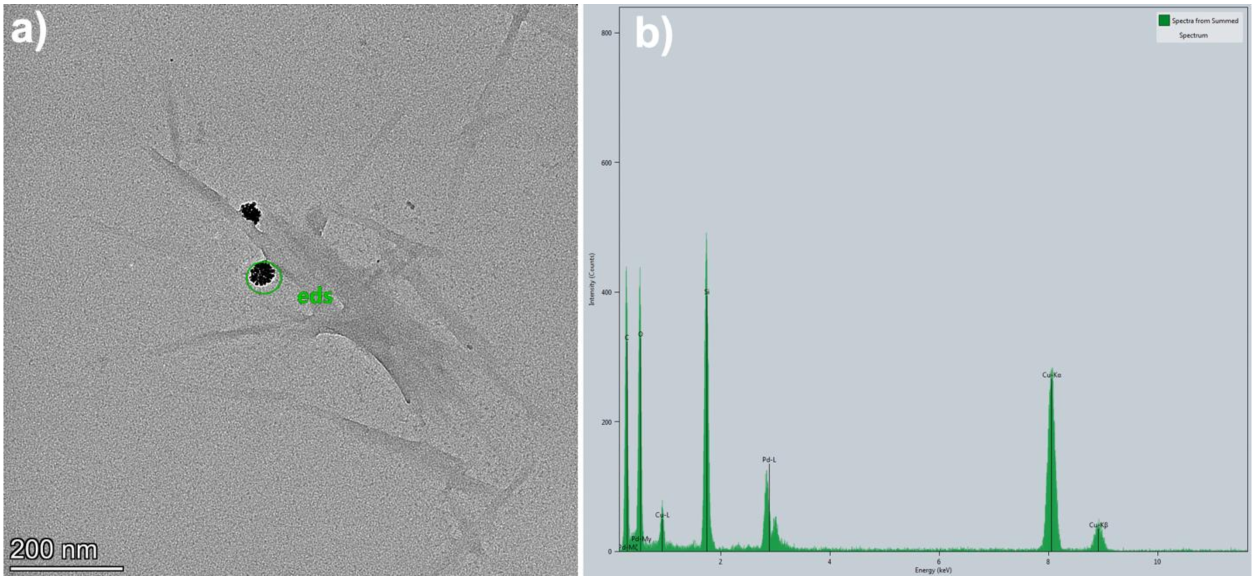

Figure S4: (a) TEM micrograph of Pd NPs deposited on ChNCs, with a green circle outlining the area in which EDX was performed. (b) Full EDX spectrum.

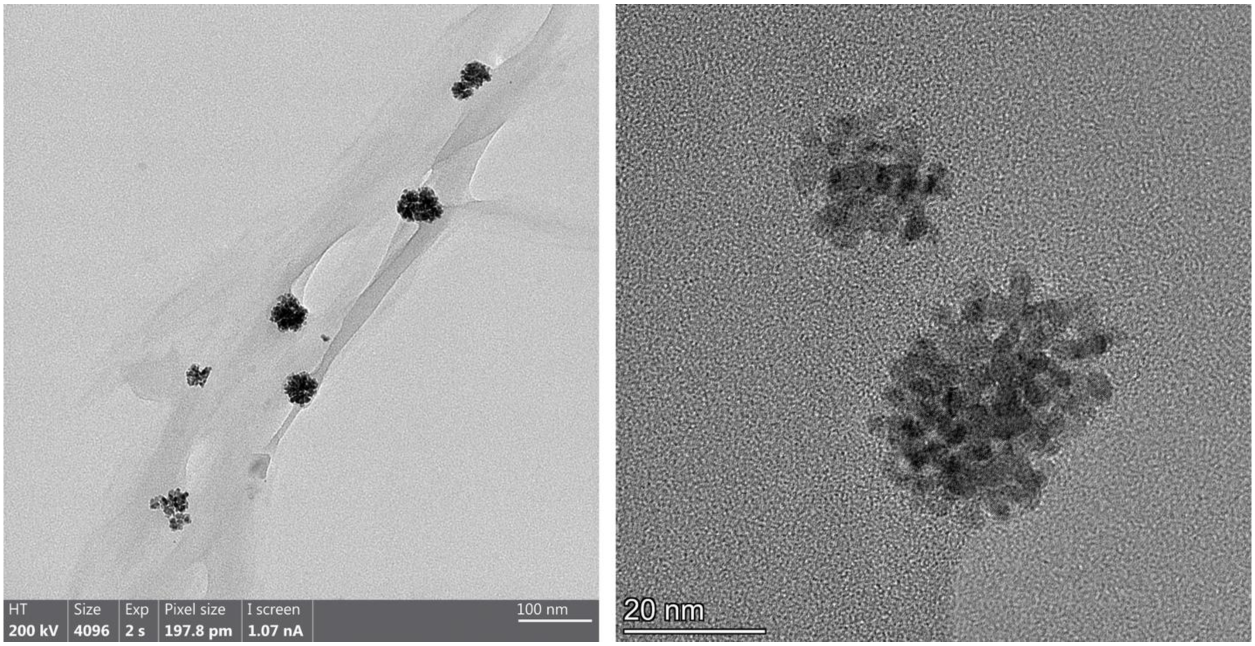

Figure S5: TEM micrographs of PdNP@ChNC at higher magnification, showing individual PdNPs agglomerated together.

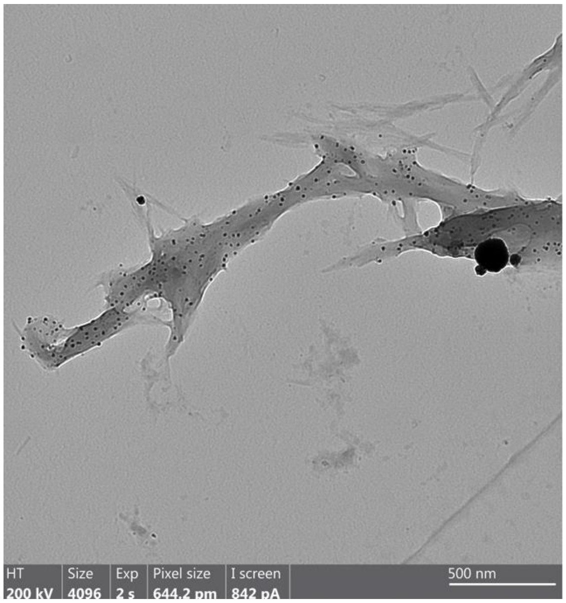

Figure S6: TEM micrograph of PdNP@ChNC with halved wt % loading of Pd relative to ChNC (0.8 wt % Pd with respect to ChNC).

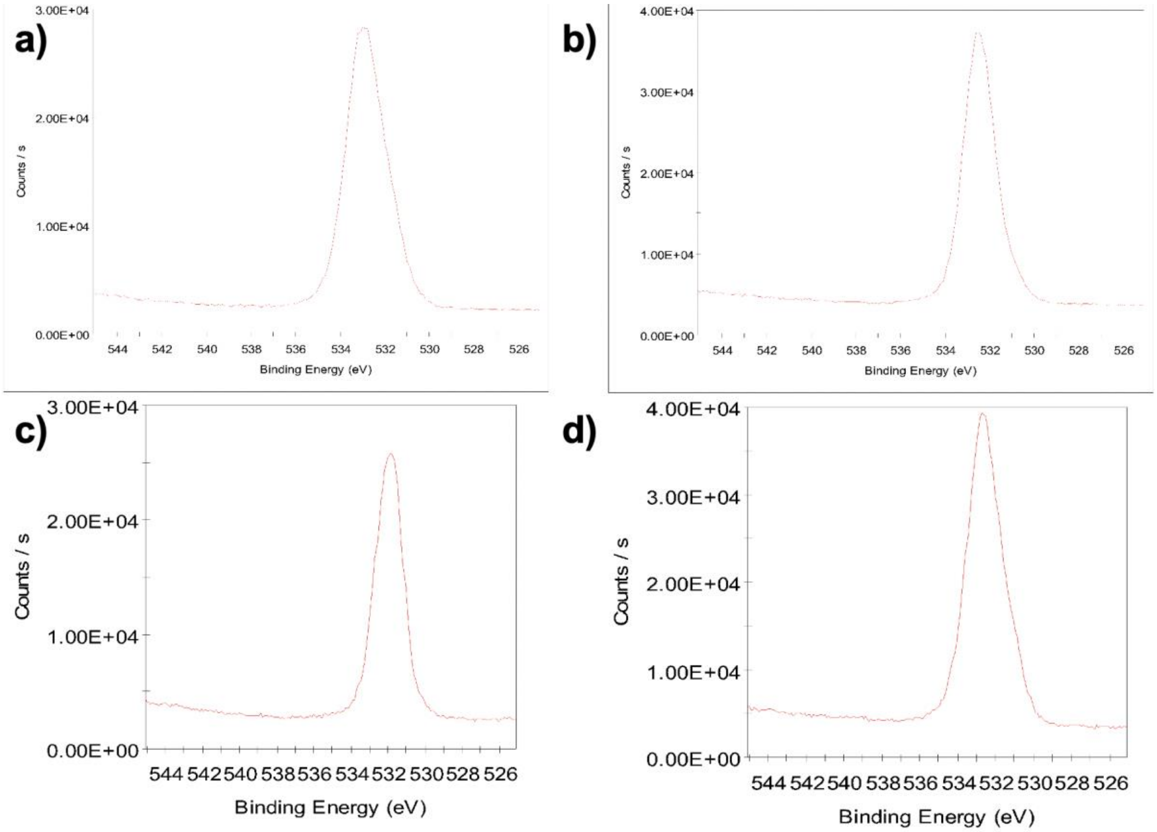

Figure S7: XPS high resolution spectra of O 1s for (a) ChNCs, (b) ChsNCs, (c) PdNP@ChNCs and (d) PdNP@ChsNCs.

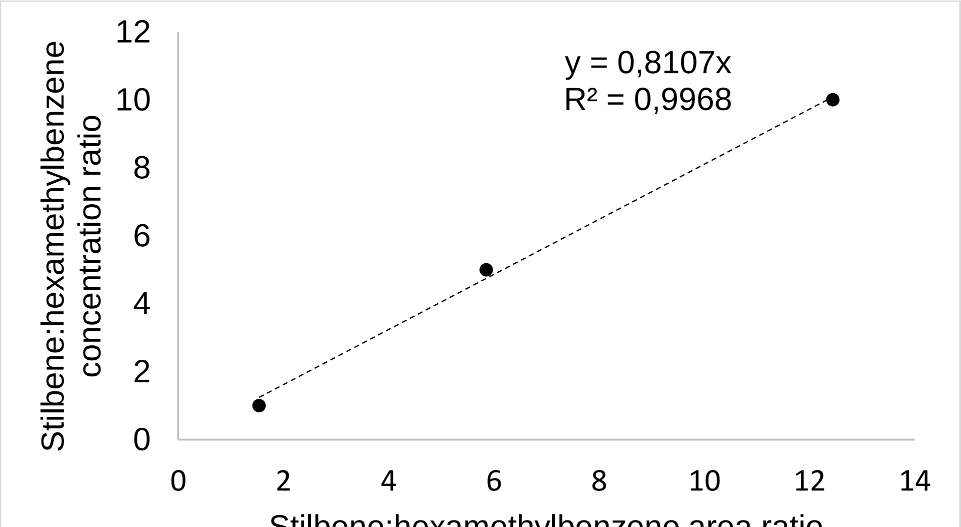

Figure S8: Calibration curve for stilbene using hexamethylbenzene as internal standard. Aliquots of 1:1, 1:5, and 1:10 of stilbene/hexamethylbenzene molar ratios were made and the subsequent corrected area ratios found were plotted.

Table S1: Representative comparison of PdNP-catalyzed Heck coupling to form stilbene found within the current literature in comparison with the PdNP@ChNC system.

Catalyst support	Catalyst loading (%)	Solvent	Temperature (°C)	Time (h)	Yield (%)	Ref
ChNC	1	1:1 v/v AcN:H_2O	90	24	100	-
CNC	0.6	1:1 v/v AcN:H_2O	100	24	75	[3]
Chitosan@SiO_2	1	DMF	110	6	92	[4]
Carbon spheres	0.5	Toluene	90	7	90	[5]
Functionalized graphene oxide	3	1:1 v/v DMF:H_2O	120	24	97	[6]
Chitin microspheres	0.6	4:1 v/v DMF:H_2O	90	10	99	[7]

References

1. Jin, T.; Kurdyla, D.; Hrapovic, S.; Leung, A. C. W.; Régnier, S.; Liu, Y.; Moores, A.; Lam, E., *Biomacromolecules* **2020,** *21*, 2236-2245.
2. Shigemasa, Y.; Matsuura, H.; Sashiwa, H.; Saimoto, H., *Int. J. Biol. Macromol.* **1996,** *18*, 237-242.
3. Cirtiu, C. M.; Dunlop-Brière, A. F.; Moores, A., *Green Chem.* **2011,** *13*, 288-291.
4. Jadhav, S.; Kumbhar, A.; Salunkhe, R., *Appl. Organomet. Chem.* **2015,** *29*, 339-345.
5. Kamal, A.; Srinivasulu, V.; Seshadri, B. N.; Markandeya, N.; Alarifi, A.; Shankaraiah, N., *Green Chem.* **2012,** *14*, 2513-2522.
6. Premi, C.; Jain, N., *RSC Adv.* **2016,** *6*, 74961-74967.
7. Pei, X.; Li, Y.; Deng, Y.; Lu, L.; Li, W.; Shi, R.; Lei, A.; Zhang, L., *Carbohydr. Polym.* **2021,** *251*, 117020.
